# Unraveling the role of blood cell perturbation responses in lung cancer by Mendelian randomization

**DOI:** 10.1007/s12672-025-02821-8

**Published:** 2025-06-15

**Authors:** Yichao Huang, Xinjing Lou, Ziqing Han, Linyu Wu, Chen Gao

**Affiliations:** 1 The First Affiliated Hospital of Zhejiang Chinese Medical University (Zhejiang Provincial Hospital of Chinese Medicine), Hangzhou, China; 2 The First School of Clinical Medicine, Zhejiang Chinese Medical University, Hangzhou, China

**Keywords:** Mendelian randomization, Lung cancer, Blood cell perturbation responses

## Abstract

**Background:**

Lung cancer remains the leading cause of cancer-related deaths globally. Emerging evidence indicates a potential link between blood cell perturbation responses and various diseases. Further investigation into the connection between these responses and lung cancer could provide valuable insights into its biological behavior and improve strategies for risk stratification.

**Methods:**

This study employs two-sample mendelian randomization, incorporating lung cancer data from the IEU OpenGWAS project, as well as hematopoietic perturbation response data, to investigate the causal relationships between hematopoietic perturbation responses and lung cancer. This study also differentiated between subtypes: small cell lung carcinoma, adenocarcinoma, and squamous cell carcinoma. The primary analytical method was the inverse variance weighted (IVW) approach. Egger intercept analysis, abnormal MR-PRESSO test, Cochran Q, and leave one out analysis were also employed as multiple sensitivity analyses to assess the robustness of the results.

**Results:**

18 blood cell perturbation responses were significantly associated with lung cancer and its subtypes, including 10 protective factors and 8 risk factors. In addition, reverse Mendelian randomization analysis identified 12 blood cells with reverse causal relationships with cancer, comprising 11 inhibitory factors and 1 promoting factor.

**Conclusions:**

Our findings demonstrate a potential causal relationship between lung cancer and blood cell perturbation responses, providing a new perspective for diagnosing and treating lung cancer. However, further studies are needed to elucidate the underlying mechanisms.

## Introduction

Lung cancer is the top cause of cancer-related deaths globally, comprising 29% of male cancer cases and 32% of female cases, and representing 20% of male and 21% of female cancer-related deaths, severely threatening human health [1]. Although research has long been conducted on lung cancer, the pathogenesis of lung cancer is a complex process involving multiple factors and remains under exploration [2, 3]. Peripheral blood is widely utilized in routine clinical testing due to its accessibility and the broad range of samples it offers, making it an ideal resource for further research. Previous studies have demonstrated a significant association between blood cell dynamics and cancer development [4–6]. For example, the qualitative or quantitative abnormalities in blood cell formation and their physiological and functional characteristics are associated with cancer susceptibility [4]. Zhou et al. also showed that platelets play an important role in cancer progression [5]. Nonetheless, the causal relationship between blood indices and cancer is still unclear [6].

While the latest research in 2024 suggests that hematological features do have a causal relationship with lung cancer [7], we still need to investigate the potential mechanisms associated with hematological features to understand the pathogenesis of lung cancer. Homilius et al. attempted to connect genetic variations and complex diseases through standardized phenotypes of primary human cells using whole blood in 2024 [8]. This approach was a departure from the previous methods of using whole genome-wideassociation studies (GWAS) with blood cell counts as the main theme [9]. However, this study did not reveal a causal relationship between lung cancer and blood cell perturbation responses.

Mendelian randomization (MR) examines potential causal relationships between exposure factors and disease outcomes, with a clear trend toward popularity [10, 11]. The drawback of observational studies is the possibility of confounding or reverse causality bias [12]. Nevertheless, MR, relying on genome-wide association studies, utilizes one or more genetic variants as instrumental variables (IVs) strongly associated with the exposure of interest and unaffected by confounders [13, 14]. In summary, although it may not be able to control for confounding and reverse causality as effectively as randomization in randomized controlled trials, MR can still help reduce bias [15].

**Fig. 1:**
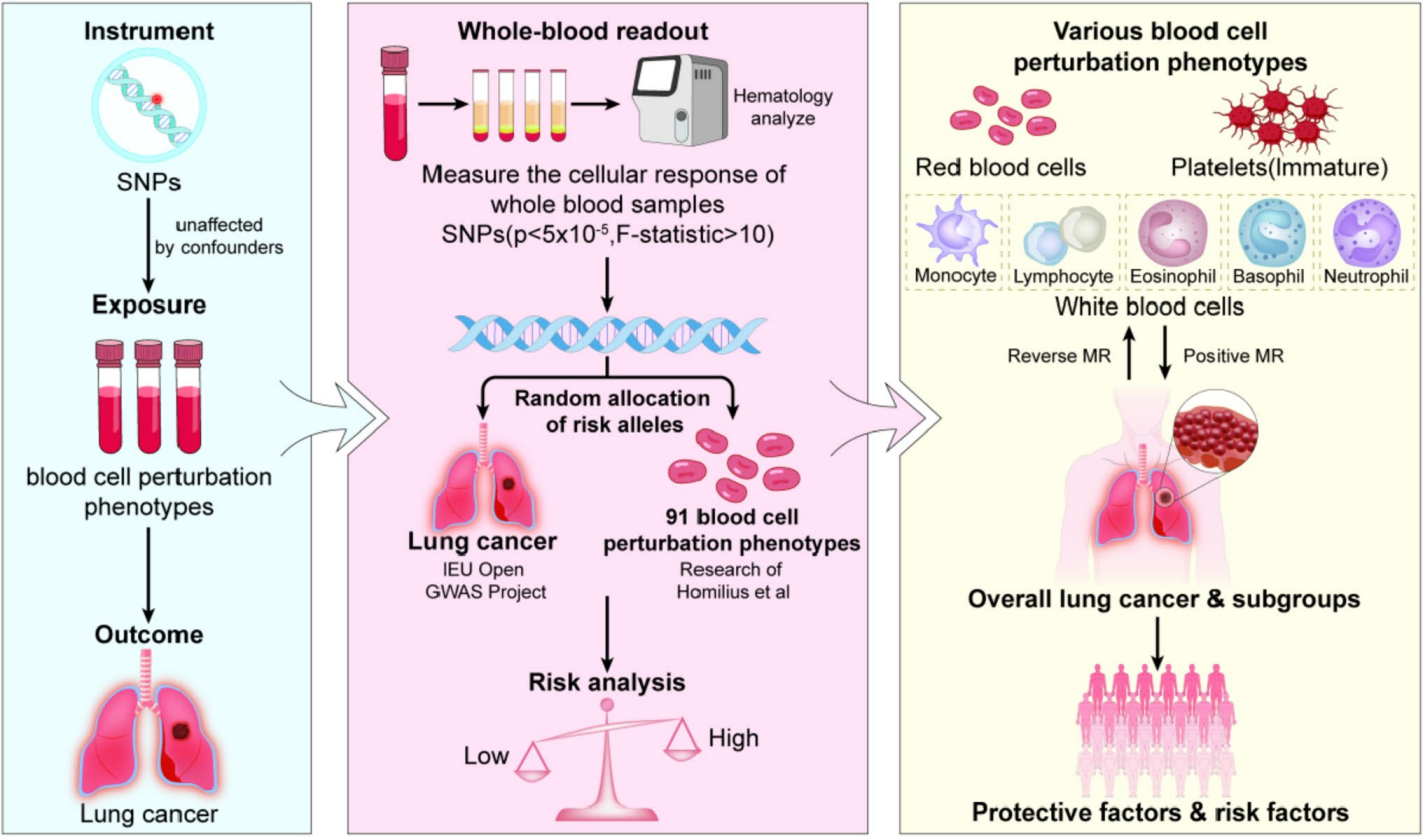
The flowchart of this study

Therefore, the present study investigates the causal relationship between blood cell perturbation responses and lung cancer through two-sample and bidirectional Mendelian randomization methods. This approach aims to provide an objective reference for the biological behavioral analysis and risk stratification of lung cancer. (Fig. 1).

## Methods

### Data sources

Homilius et al. conducted genome-wide analysis on perturbed blood cell phenotypes, combined with functional readings based on flow cytometry, to identify various locus data related to latent cell traits [8]. After filtering and removing linkage disequilibrium, we obtained 91 different blood cell perturbed phenotype data. We adopted the “cell type perturbation response (Channel, Condition, Readout)” format for naming the corresponding phenotypes. These channels were mainly divided into four fluorescent dyes, including white cell differential channel by fluorescence (WDF) dye, white count and nucleated red blood cells (WNR), reticulocyte (RET), and platelet-F (PLT-F). Readouts were mainly divided into side scatter (SSC), forward scatter (FSC), and side fluorescence (SFL).

We acquired the corresponding genetic association data for lung cancer from the IEU Open GWAS Project (https://gwas.mrcieu.ac.uk/) and ultimately obtained five datasets as the exploration outcome. The overall lung cancer-related GWAS IDs were ukb-b-20176 and ukb-b-14521, while the GWAS IDs of non-small cell lung carcinoma (NSCLC) subtypes squamous cell lung cancer and lung adenocarcinoma were ieu-a-989 and ieu-a-984, respectively. The GWAS ID for small cell lung carcinoma (SCLC) is ieu-a-988. The specific data of the dataset can be found in Table 1.

**Table 1:** Specific information of lung cancer dataset

Phenotype	GWAS ID	Sample size (cases/controls)	Population	Build
Lung cancer	ukb-b-20176	17,566/405,692	European	HG19/GRCh37
Lung cancer	ukb-b-14521	37,443/364,181	European	HG19/GRCh37
Squamous cell lung cancer	ieu-a-989	7704/54,763	European	HG19/GRCh37
Small cell lung carcinoma	ieu-a-988	2791/20,580	European	HG19/GRCh37
Lung adenocarcinoma	ieu-a-984	11,245/54,619	European	HG19/GRCh37

### Selection criteria

We filtered out single nucleotide polymorphisms (SNPs) with *P* > 5 × 10^–5^ to select highly significant instrumental variables and removed linkage disequilibrium. This threshold can increase the number of instrumental variables, thereby enhancing statistical power. Moreover, a greater number of instrumental variables can better capture the causal relationship between exposure and outcome [16]. The above linkage disequilibrium was defined as having R^2^ > 0.001 and within a physical distance of 10,000 kb [17]. Only SNPs with an F-statistic greater than ten were selected to ensure that the selected SNPs have sufficient statistical power [18].

### Mendelian randomization analysis

We conducted Mendelian randomization analysis on R software (version 4.4.1) using the R package "TwoSampleMR" (version 0.6.6). Given that the currently most widely recognized method for MR analysis is the inverse variance weighted (IVW) method, the statistical analysis here mainly uses IVW as the main evaluation method [19]. To account for multiple testing correction, we employed the False Discovery Rate (FDR) method for multiplicity adjustment, with the significance threshold set at 0.05 [20]. Only results with a *P* < 0.05/91 = 5.5 × 10^–4^ were considered significant. In addition, we also added MR Egger, weighted median, simple mode, and weighted mode methods to improve the accuracy of the evaluation [21].

### Sensitivity analysis

To confirm that the observed association between exposure and outcome was not influenced by confounding factors, such as SNPs affecting traits through independent pathways (horizontal pleiotropy), we performed pleiotropy analyses. These included the MR-Egger intercept analysis and the MR-PRESSO outlier test. The MR-PRESSO test was performed with 1000 simulations to ensure the stability and reliability of the results. No evidence of horizontal pleiotropy was detected, as indicated by *P*-values greater than 0.05 [22, 23]. We also conducted the Cochran Q test to avoid heterogeneity and removed outlier sets [22]. On this basis, we further conducted a Leave-One-Out analysis to ensure the stability of the results. The mr function was used for Mendelian Randomization analysis. The mr_pleiotropy_test function was used for MR-Egger intercept analysis, while the run_mr_presso function was used for the MR-PRESSO test. The mr_heterogeneity function was used for heterogeneity testing. The mr_leaveoneout function was employed for Leave-One-Out analysis.

Genetic variation influencing exposure through outcome may lead to erroneous inferences about causality, especially in complex biological processes or interacting factors [24]. To avoid the effects of reverse causality and confounding factors on the results, we performed a bidirectional Mendelian randomization analysis, with filtered cancer datasets as exposure factors and different blood cell perturbation responses as outcomes. *P* < 0.05 was considered statistically significant.

### Confounding factor analysis

To assess the potential horizontal pleiotropy of the MR results, we conducted several sensitivity analyses to identify SNPs that may violate the MR assumptions. Nevertheless, some pleiotropic SNPs that are difficult to detect may still exist. To further evaluate whether each SNP is associated with established risk factors for lung cancer, such as smoking, we employed the LDtrait method to account for potential confounding factors [25, 26]. We excluded the SNPs that were significantly associated with confounding factors and conducted the MR analysis again. In this way, we ensure the accuracy and robustness of the MR analysis.

## Results

### The causal relationship between blood cell perturbation responses and lung cancer

**Fig. 2:**
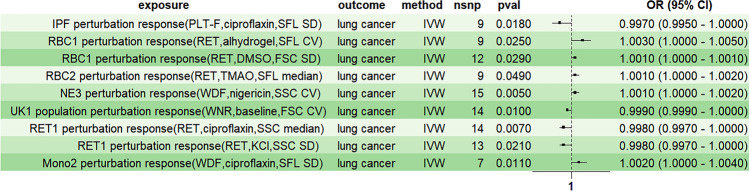
Univariate Mendelian randomization forest plot analysis of blood perturbation responses and overall lung cancer risk. *IVW* inverse variance weighting, *CV* coefficient of variation, *FSC* forward scatter, *PLT-F* platelet-F, *RET* reticulocyte, *SD* standard deviation, *SFL* side fluorescence, *SSC* side scatter, *WDF* white cell differential channel by fluorescence, *WNR* white count and nucleated red blood cells

To assess the causal relationship between blood cell perturbation responses and overall lung cancer, we first conducted univariate Mendelian Randomization (MR) analysis. The inverse-variance weighted (IVW) method was used as the primary analytical approach to screen nine blood cell perturbation responses (*P* < 0.05, Table 2, Fig. 2).

**Table 2:** Causal effects of blood perturbation responses on overall lung cancer

Exposure	Outcome	nsnp	*P*-value	Odds ratio	OR_lci95	OR_uci95
IPF perturbation response (PLT-F, ciprofloxacin, SFL SD)	Lung cancer	9	0.018	0.997	0.995	1.000
RBC1 perturbation response (RET, alhydrogel, SFL CV)	Lung cancer	9	0.025	1.003	1.000	1.005
RBC1 perturbation response (RET, DMSO, FSC SD)	Lung cancer	12	0.029	1.001	1.000	1.001
RBC2 perturbation response (RET, TMAO, SFL median)	Lung cancer	9	0.049	1.001	1.000	1.002
NE3 perturbation response (WDF, nigericin, SSC CV)	Lung cancer	15	0.005	1.001	1.000	1.002
UK1 population perturbation response (WNR, baseline, FSC CV)	Lung cancer	14	0.010	0.999	0.999	1.000
RET1 perturbation response (RET, ciprofloxacin, SSC median)	Lung cancer	14	0.007	0.998	0.997	1.000
RET1 perturbation response (RET, KCl, SSC SD)	Lung cancer	13	0.021	0.998	0.997	1.000
Mono2 perturbation response (WDF, ciprofloxacin, SFL SD)	Lung cancer	7	0.011	1.002	1.000	1.004

*CV* coefficient of variation, *FSC* forward scatter, *PLT-F* platelet-F, *RET* reticulocyte, *SD* standard deviation, *SFL* side fluorescence, *SSC* side scatter, *WDF* white cell differential channel by fluorescence, *WNR* white count and nucleated red blood cells, *OR_lci95* lower 95% confidence interval odds ratio, *OR_uci95* upper 95% confidence interval odds ratio

The analysis showed that certain blood cell perturbation responses were associated with a reduced risk of lung cancer. The immature platelet fraction (IPF) perturbation response (PLT-F, ciprofloxacin, SFL SD) had an odds ratio (OR) of 0.997 (*P*: 0.018, 95% CI: 0.995–1.000). Similarly, the unknown cell (UK)1 population perturbation response (WNR, baseline, FSC CV) demonstrated an OR of 0.999 (*P*: 0.010, 95% CI: 0.999–1.000). Additionally, the reticulocyte (RET)1 perturbation response (RET, ciprofloxacin, SSC median) had an OR of 0.998 (*P*: 0.007, 95% CI: 0.997–1.000), and the RET1 perturbation response (RET, KCl, SSC SD) had an OR of 0.998 (*P*: 0.021, 95% CI: 0.997–1.000).

Conversely, other blood cell perturbation responses were associated with an increased risk of lung cancer. The red blood cells (RBC)1 perturbation response (RET, alhydrogel, SFL CV) had an OR of 1.003 (*P*: 0.025, 95% CI: 1.000–1.005), while another RBC1 perturbation response (RET, DMSO, FSC SD) had an OR of 1.001 (*P*: 0.029, 95% CI: 1.000–1.001). The RBC2 perturbation response (RET, TMAO, SFL median) showed an OR of 1.001 (*P*: 0.049, 95% CI: 1.000–1.002). In addition, the neutrophils (NE3) perturbation response (WDF, nigericin, SSC CV) was associated with an OR of 1.001 (*P*: 0.005, 95% CI: 1.000–1.002), and the monocytes (Mono)2 perturbation response (WDF, ciprofloxacin, SFL SD) had an OR of 1.002 (*P*: 0.011, 95% CI: 1.000–1.004).

These findings can be grouped into six overarching blood cell perturbation responses, including those involving IPF, RBC, RET, Mono, NE, and UK populations. Although the overall impact of blood cell perturbation responses on lung cancer risk is not statistically significant, these results suggest a correlation between blood cell perturbation responses and lung cancer occurrence, warranting further investigation.

**Fig. 3:**
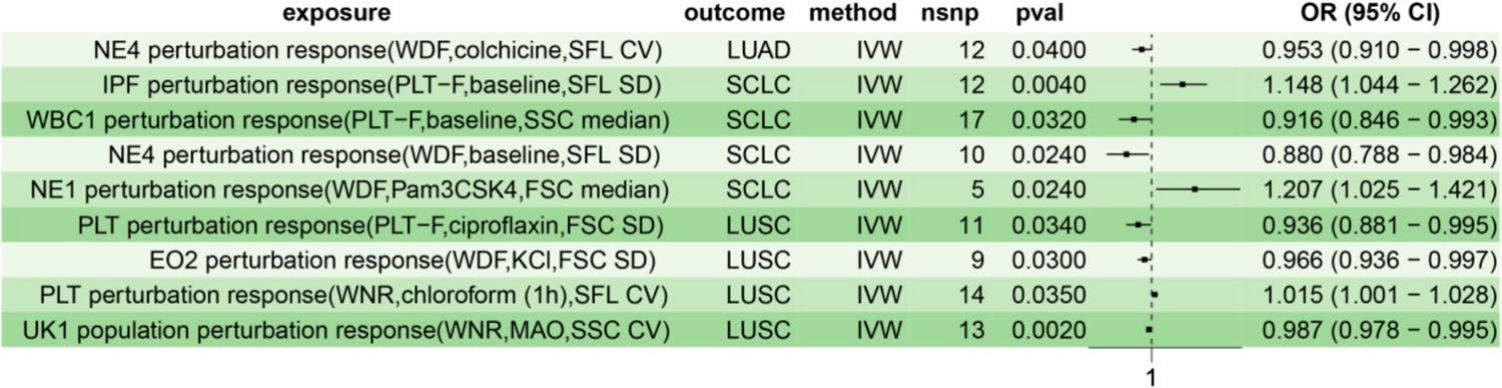
Univariate Mendelian randomization forest plot analysis of blood perturbation responses and subtypes of lung cancer. *IVW* inverse variance weighting, *LUAD* lung adenocarcinoma, *SCLC* small cell lung carcinoma, *LUSC* squamous cell lung cancer

To further clarify the causal effects of blood cell perturbation response on lung cancer, we mainly conducted Mendelian randomization analysis on the most common subtypes of non-small and small cell lung carcinoma. Non-small cell lung carcinoma was further divided into subtypes of lung adenocarcinoma and squamous cell lung cancer as outcome variables. We identified nine distinct blood cell perturbation responses associated with various lung cancer subtypes (Table 3, Fig. 3). The NE4 perturbation response (WDF, colchicine, SFL CV) was associated with a reduced risk of lung adenocarcinoma (OR: 0.953, *P*: 0.040, 95% CI: 0.910–0.998). For squamous cell lung cancer, the PLT perturbation response (PLT-F, ciprofloxacin, FSC SD; OR: 0.936, *P*: 0.034, 95% CI: 0.881–0.995), eosinophils (EO)2 perturbation response (WDF, KCl, FSC SD; OR: 0.966, *P*: 0.030, 95% CI: 0.936–0.997), and UK population perturbation response (WNR, MAO, SSC CV; OR: 0.987, *P*: 0.002, 95% CI: 0.995) were found to reduce risk. However, the PLT perturbation response (WNR, chloroform (1 h), SFL CV) increased the risk of squamous cell lung cancer (OR: 1.015, *P*: 0.035, 95% CI: 1.001–1.028). Regarding small cell lung carcinoma, the white blood cell (WBC)1 perturbation response (PLT-F, baseline, SSC median) reduced risk (OR: 0.916, *P*: 0.032, 95% CI: 0.846–0.993), as did the NE4 perturbation response (WDF, baseline, SFL SD; OR: 0.880, *P*: 0.024, 95% CI: 0.788–0.984). In contrast, the IPF perturbation response (PLT-F, baseline, SFL SD) increased risk (OR: 1.148, *P*: 0.004, 95% CI: 1.044–1.262), as did the NE1 perturbation response (WDF, Pam3CSK4, FSC median; OR: 1.207, *P*: 0.024, 95% CI: 1.025–1.421). Based on the above results, the cell perturbation responses of EO and WBC were uniquely associated with squamous cell and small cell lung carcinoma, respectively, compared to overall lung cancer. However, the RBC perturbation response, RET perturbation response, and Mono perturbation response were associated with overall lung cancer and not specific subtypes. After FDR correction, all results regarding the perturbation response of blood cells were negative.

**Table 3:** Causal effects of blood perturbation responses on subtypes of lung cancer

Exposure	Outcome	nsnp	*P*-value	Odds ratio	OR_lci95	OR_uci95
NE4 perturbation response (WDF, colchicine, SFL CV)	Lung adenocarcinoma	12	0.040	0.953	0.910	0.998
IPF perturbation response (PLT-F, baseline, SFL SD)	Small cell lung carcinoma	12	0.004	1.148	1.044	1.262
WBC1 perturbation response (PLT-F, baseline, SSC median)	Small cell lung carcinoma	17	0.032	0.916	0.846	0.993
NE4 perturbation response (WDF, baseline, SFL SD)	Small cell lung carcinoma	10	0.024	0.880	0.788	0.984
NE1 perturbation response (WDF, Pam3CSK4, FSC median)	Small cell lung carcinoma	5	0.024	1.207	1.025	1.421
PLT perturbation response (PLT-F, ciprofloxacin, FSC SD)	Squamous cell lung cancer	11	0.034	0.936	0.881	0.995
EO2 perturbation response (WDF, KCl, FSC SD)	Squamous cell lung cancer	9	0.030	0.966	0.936	0.997
PLT perturbation response (WNR, chloroform (1 h), SFL CV)	Squamous cell lung cancer	14	0.035	1.015	1.001	1.028
UK1 population perturbation response (WNR, MAO, SSC CV)	Squamous cell lung cancer	13	0.002	0.987	0.978	0.995

**Fig. 4:**
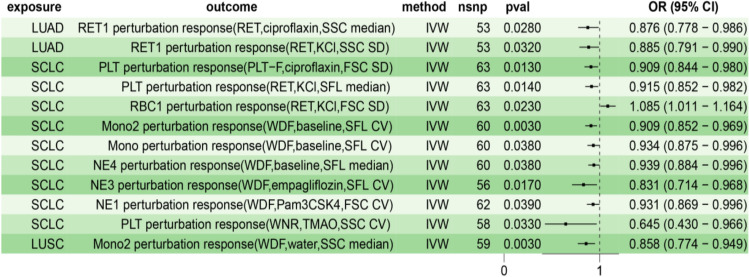
Reverse Mendelian randomization forest plot analysis of blood perturbation responses and lung cancer. *IVW* inverse variance weighting, *LUAD* lung adenocarcinoma, *SCLC* small cell lung carcinoma, *LUSC* squamous cell lung cancer

Subsequently, we conducted a reverse MR analysis and identified 12 blood cell perturbation responses that may be caused by lung cancer (Table 4, Fig. 4). It is worth noting that lung cancer does not show a clear reverse causal relationship with blood cell perturbation responses. However, squamous cell lung cancer reduced the risk of Mono2 perturbation response. Small cell lung carcinoma reduced the risk of PLT, Mono, and NE perturbation responses and increased the risk of RBC perturbation response. Lung adenocarcinoma reduced the risk of RET1 perturbation response. Another noteworthy point is that the disturbance response of RET may reduce the overall risk of lung cancer while also being suppressed by lung adenocarcinoma.

**Table 4:** Causal effects of lung cancer and its subtypes on blood perturbation responses

Exposure	Outcome	nsnp	*P*-value	Odds ratio	OR_lci95	OR_uci95
Lung adenocarcinoma	RET1 perturbation response (RET, ciprofloxacin, SSC median)	53	0.028	0.876	0.778	0.986
Lung adenocarcinoma	RET1 perturbation response (RET, KCl, SSC SD)	53	0.032	0.885	0.791	0.990
Small cell lung carcinoma	PLT perturbation response (PLT-F, ciprofloxacin, FSC SD)	63	0.013	0.909	0.844	0.980
Small cell lung carcinoma	PLT perturbation response (RET, KCl, SFL median)	63	0.014	0.915	0.852	0.982
Small cell lung carcinoma	RBC1 perturbation response (RET, KCl, FSC SD)	63	0.023	1.085	1.011	1.164
Small cell lung carcinoma	Mono2 perturbation response (WDF, baseline, SFL CV)	60	0.003	0.909	0.852	0.969
Small cell lung carcinoma	Mono perturbation response (WDF, baseline, SFL CV)	60	0.038	0.934	0.875	0.996
Small cell lung carcinoma	NE4 perturbation response (WDF, baseline, SFL median)	60	0.038	0.939	0.884	0.996
Small cell lung carcinoma	NE3 perturbation response (WDF, empagliflozin, SFL CV)	56	0.017	0.831	0.714	0.968
Small cell lung carcinoma	NE1 perturbation response (WDF, Pam3CSK4, FSC CV)	62	0.039	0.931	0.869	0.996
Small cell lung carcinoma	PLT perturbation response (WNR, TMAO, SSC CV)	58	0.033	0.645	0.430	0.966
Squamous cell lung cancer	Mono2 perturbation response (WDF, water, SSC median)	59	0.003	0.858	0.774	0.949

### Sensitivity analyses

Several sensitivity analyses were conducted to test the accuracy of the MR analysis, including Cochran's Q test, MR Egger intercept test, MR-PRESSO test, and Leave-One-Out test. The MR Egger intercept test showed that all the screened blood cell perturbation responses had no horizontal pleiotropy (*P* > 0.05). After correcting for outliers through MR-PRESSO analysis (*P* > 0.05). The Cochran's Q test showed no significant difference between different blood cell perturbation responses and lung cancer (*P* > 0.05).In addition, the Leave-One-Out test also showed stability. Therefore, the MR analysis results were reliable. The results of Cochran's Q test, MR Egger intercept test, and MR-PRESSO test are shown in Table 5.

**Table 5:** MR analysis multiple sensitivity analysis results

Exposure	Outcome	MR-Egger intercept test		MR-PRESSO	Cochran’s Q test	
		Egger_intercept	*P*-value	*P*-value	Q	Q_pval
IPF perturbation response (PLT-F, ciproflaxin, SFL SD)	Lung cancer	−0.001	0.520	0.899	3.487	0.900
RBC1 perturbation response (RET, alhydrogel, SFL CV)	Lung cancer	−0.001	0.464	0.332	9.957	0.268
RBC1 perturbation response (RET, DMSO, FSC SD)	Lung cancer	0.001	0.503	0.852	6.521	0.836
RBC2 perturbation response (RET, TMAO, SFL median)	Lung cancer	0.000	0.762	0.599	6.794	0.559
NE3 perturbation response (WDF, nigericin, SSC CV)	Lung cancer	0.000	0.957	0.855	8.189	0.879
UK1 population perturbation response (WNR, baseline, FSC CV)	Lung cancer	0.000	0.791	0.935	6.541	0.924
RET1 perturbation response (RET, ciprofloxacin, SSC median)	Lung cancer	0.000	0.676	0.558	11.947	0.532
RET1 perturbation response (RET, KCl, SSC SD)	Lung cancer	0.001	0.056	0.337	14.030	0.299
Mono2 perturbation response (WDF, ciprofloxacin, SFL SD)	Lung cancer	0.000	0.894	0.682	4.597	0.596
NE4 perturbation response (WDF, colchicine, SFL CV)	Lung adenocarcinoma	0.003	0.900	0.872	6.200	0.860
IPF perturbation response (PLT-F, baseline, SFL SD)	Small cell lung carcinoma	−0.036	0.214	0.755	7.631	0.746
WBC1 perturbation response (PLT-F, baseline, SSC median)	Small cell lung carcinoma	−0.026	0.425	0.436	16.890	0.393
NE4 perturbation response (WDF, baseline, SFL SD)	Small cell lung carcinoma	0.017	0.674	0.537	8.426	0.492
NE1 perturbation response (WDF, Pam3CSK4, FSC median)	Small cell lung carcinoma	0.100	0.242	0.619	2.787	0.594
PLT perturbation response (PLT-F, ciprofloxacin, FSC SD)	Squamous cell lung cancer	−0.036	0.266	0.519	9.556	0.480
E2 perturbation response (WDF, KCl, FSC SD)	Squamous cell lung cancer	−0.015	0.684	0.841	4.293	0.830
PLT perturbation response (WNR, chloroform (1 h), SFL CV)	Squamous cell lung cancer	0.025	0.342	0.739	9.703	0.718
UK1 population perturbation response (WNR, MAO, SSC CV)	Squamous cell lung cancer	0.015	0.595	0.470	12.062	0.441

**Fig. 5:**
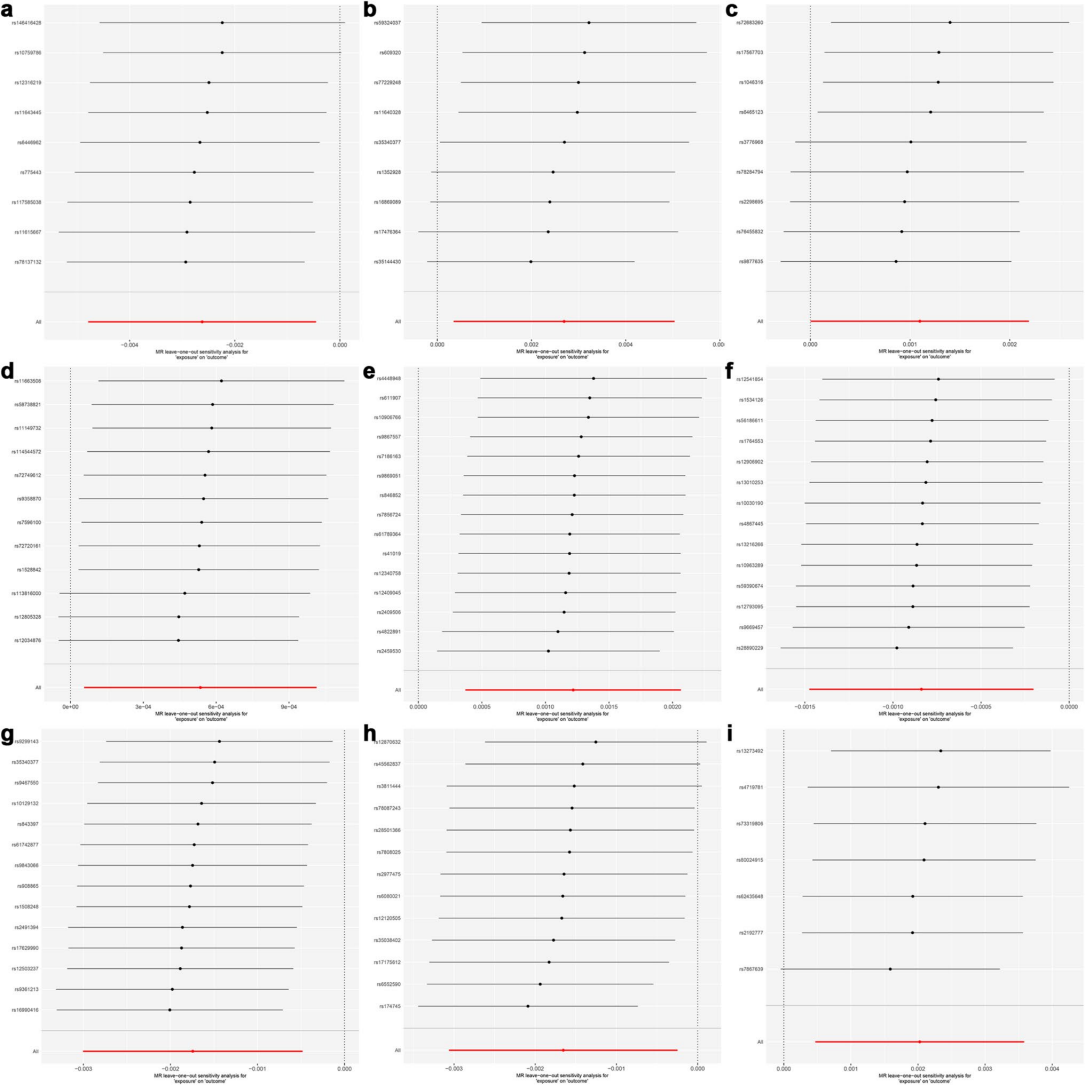
Leave-One-Out experimental results for MR analysis of the causal relationship between blood perturbation responses and overall lung cancer. **a** IPF perturbation response (PLT-F, ciprofloxacin, SFL SD), **b** RBC1 perturbation response (RET, alhydrogel, SFL CV), **c** RBC1 perturbation response (RET, DMSO, FSC SD), **d** RBC2 perturbation response (RET, TMAO, SFL median), **e** NE3 perturbation response (WDF, nigericin, SSC CV), **f** UK1 population perturbation response (WNR, baseline, FSC CV), **g** RET1 perturbation response (RET, ciprofloxacin, SSC median), **h** RET1 perturbation response (RET, KCl, SSC SD), **i** Mono2 perturbation response (WDF, ciprofloxacin, SFL SD)

For the reverse MR analysis of lung cancer and blood cell perturbation responses, the MR-PRESSO results and MR Egger intercept results showed that the data had no pleiotropy (*P* > 0.05). Cochran's Q test also showed low heterogeneity levels, ensuring the results' robustness (*P* > 0.05). In addition, Leave-One-Out analysis once again provides the stability of the results (Fig. 5). In summary, the results of reverse MR analysis are also reliable. The results of Cochran's Q test, MR Egger intercept test, and MR-PRESSO test are shown in Table 6.

**Table 6:** Reverse MR analysis multiple sensitivity analysis results

Exposure	Outcome	MR-Egger intercept test		MR-PRESSO	Cochran’s Q test	
		Egger_intercept	*P*-value	*P*-value	Q	Q_pval
Lung adenocarcinoma	RET1 perturbation response (RET, ciprofloxacin, SSC median)	0.000	0.997	0.348	55.716	0.337
Lung adenocarcinoma	RET1 perturbation response (RET, KCl, SSC SD)	−0.034	0.064	0.609	47.611	0.647
Small cell lung carcinoma	PLT perturbation response (PLT-F, ciprofloxacin, FSC SD)	−0.002	0.934	0.260	68.973	0.253
Small cell lung carcinoma	PLT perturbation response (RET, KCl, SFL median)	0.001	0.982	0.831	51.443	0.828
Small cell lung carcinoma	RBC1 perturbation response (RET, KCl, FSC SD)	−0.025	0.320	0.686	56.267	0.681
Small cell lung carcinoma	Mono2 perturbation response (WDF, baseline, SFL CV)	0.005	0.842	0.992	36.234	0.991
Small cell lung carcinoma	Mono perturbation response (WDF, baseline, SFL CV)	0.021	0.382	0.912	44.388	0.921
Small cell lung carcinoma	NE4 perturbation response (WDF, baseline, SFL median)	0.004	0.840	0.928	43.591	0.933
Small cell lung carcinoma	NE3 perturbation response (WDF, empagliflozin, SFL CV)	−0.097	0.097	0.943	39.264	0.946
Small cell lung carcinoma	NE1 perturbation response (WDF, Pam3CSK4, FSC CV)	−0.006	0.824	0.898	47.448	0.898
Small cell lung carcinoma	PLT perturbation response (WNR, TMAO, SSC CV)	0.096	0.512	0.427	57.897	0.442
Squamous cell lung cancer	Mono2 perturbation response (WDF, water, SSC median)	0.007	0.691	0.559	56.636	0.526

## Discussion

GWAS technology is useful for understanding the genetic basis of complex diseases. However, it difficult to relate genetic and biological effects [27]. Our research underscores the potential of cytogenetic perturbation in identifying novel cell populations and diverse blood cell perturbation responses that may act as biomarkers for disease states and guide treatment strategies [28]. In this study, building on the work of Homilius et al., we employed Mendelian Randomization to investigate the causal relationship between 91 blood cell perturbation responses and lung cancer.

Initially, we identified nine blood cell perturbation responses associated with overall lung cancer, comprising four protective factors and five risk factors. Further analysis focused on lung cancer subtypes, identifying nine additional blood cell perturbation responses, including six protective factors and three risk factors. Moreover, reverse MR analysis revealed 12 blood cell responses with reverse causality to lung cancer, encompassing 11 inhibitory factors and one promoting factor. These findings highlight the intricate relationship between blood cell perturbation responses and lung cancer, offering new insights into potential biomarkers and therapeutic targets. Despite the FDR correction, all results of the blood cell perturbation response were negative. However, in some preliminary screening stages, overly stringent FDR correction may lead to the omission of some potentially significant findings [29]. In our study, only results with a *P* < 0.05/91 = 5.5 × 10^–4^ were considered significant, which is evidently overly stringent. Therefore, we regard the FDR-corrected results as a reference rather than the definitive explanation of the final results.

As early as 2015, reticulocyte counting was applied to cancer diagnosis by identifying anemia in cancer patients [30]. Recent studies further indicate that the level of reticulocytes in blood parameters is associated with the prognosis of various malignant tumors [31]. Research has revealed that anemia frequently co-occurs with cancer, and the reticulocyte count serves as a valuable diagnostic tool for this condition [32, 33]. Moreover, the presence and severity of anemia are closely correlated with the stage of cancer [34]. This may be because anemia can further exacerbate the hypoxic state of the tumor microenvironment, thereby promoting tumor invasion and metastasis [35]. However, in hypoxic conditions, the increase in mitochondria in reticulocytes can generate ROS, and the high levels of ROS can inhibit cancer cells, which may be one of the mechanisms by which the RET perturbation response can reduce the risk of lung cancer [36, 37]. Additionally, it has been demonstrated that reticulocytes, particularly those induced under stress conditions, contain and/or express components that can enhance immune function. This may also be one of the mechanisms through which the RET perturbation response reduces the risk of lung cancer [38]. Our research suggests that RET perturbation responses triggered by different stressors reduce the risk of overall lung cancer. This indicates that the RET perturbation response may play a crucial role in enabling reticulocytes to prevent or treat cancer.

Contrary to the RET perturbation response, the RBC perturbation response increased the overall risk of lung cancer. Previous studies have found that RBCs and their derivatives play a crucial role in developing lung cancer [39]. Red blood cells may contain DNA fragments and absorb DNA carrying oncogenic mutations from cancer cells or tissues through intercellular contact [40]. In addition, RBC miRNA can potentially serve as a novel biomarker for lung cancer [39]. Research has further indicated that the unique interactions between red blood cells and tumor cells also facilitate the progression of tumor cells [41]. Moreover, the immunosuppressive functions of red blood cells, which can be induced by metabolites, can also be initiated within the tumor microenvironment, thereby playing a crucial role in the development and progression of cancer [42]. The RBC perturbation response may facilitate this process, potentially increasing the risk associated with lung cancer. The results of this study further highlight the role of red blood cells in promoting cancer development, with the RBC perturbation response potentially being a key factor in their influence on lung cancer onset. In summary, both the RET and RBC perturbation responses have distinct impacts on the development of lung cancer. Previous studies have shown many highly significant interactions between monocyte-derived macrophages (mo-Macs) and invasive/metastatic tumor cells [43]. Our results show that Mono perturbation response increased the risk of overall lung cancer, which also indirectly confirms this.

Furthermore, White blood cells perturbation responses can significantly reduce the risk of developing SCLC. Given WBC’s immune role involvement, this result aligns with expectations [44]. However, the WBC perturbation response did not show significant effects on NSCLC. Although the literature has pointed out that salivary lipids in NSCLC patients exhibit disturbances to plasma, they mainly target plasma lipids [45]. In addition, WBC can be divided into granulocytes and agranulocytes [46]. Previous studies have shown that eosinophils are important in anti-cancer immunity [47]. Moreover, eosinophils are involved in various homeostatic processes, suggesting that these cells may play a critical role in metabolic regulation and organ function in healthy individuals [48]. The results of our study confirm that EO perturbation response can specifically reduce the risk of Squamous cell lung cancer.

Neutrophils are an important component of cancer immune infiltration and are known to accumulate in various types of cancer [49, 50]. However, their heterogeneous nature allows them to play distinct roles in cancer progression and treatment [51]. Our research also supports this variability, which demonstrates that the NE perturbation response promotes overall lung cancer development but inhibits lung adenocarcinoma. This functional heterogeneity is particularly pronounced in small-cell lung cancer, where one response promotes progression while another inhibits it. The biological mechanisms underlying these diverse functional states remain largely unclear [52].

Furthermore, reverse MR shows that lung adenocarcinoma significantly reduces the risk of RET perturbation response, while RET perturbation response can reduce the overall risk of lung cancer. Indicating that RET perturbation response may have unique preventive and therapeutic effects on lung cancer. However, the occurrence of lung adenocarcinoma can inhibit its effects. The underlying mechanism may be that lung adenocarcinoma-induced cancer-related anemia activates the mechanisms of stress erythropoiesis, characterized by an enlarged pool of erythroid progenitor cells, low reticulocyte count, and largely unable to differentiate and produce mature red blood cells [53]. Reverse MR also showed that SCLC promotes RBC perturbation response. Under the premise that the RBC disturbance response increases the overall risk of lung cancer, there appears to be a potential malignant causal cycle between the RBC disturbance response and small-cell lung cancer. This cycle may be driven by the tumor burden of small-cell lung cancer, which reduces the relative deformability of red blood cells, thereby impairing their function [54].

While both SCLC and NSCLC involve interactions with platelets, the nature of these interactions may differ based on the distinct biological characteristics of each cancer type. For example, SCLC is characterized by rapid proliferation and early metastasis, which is different from NSCLC and may involve distinct platelet-mediated mechanisms [55]. In our study, SCLC exerts a pronounced inhibitory effect on the PLT perturbation response, whereas NSCLC did not demonstrate this characteristic. This may be attributed to the fact that, compared with patients with NSCLC, the platelet count in SCLC patients is negatively correlated with the absolute amount of cyclophosphamide and doxorubicin received, and platelet counts decline significantly with disease progression and mortality, which undoubtedly affects the role of the PLT perturbation response [56]. Moreover, SCLC significantly suppresses the NE perturbation response, a feature not observed in NSCLC. However, due to the heterogeneity of neutrophils in cancer progression, the clinical applicability of the NE perturbation response remains uncertain [57].

In fact, although both adenocarcinoma and squamous cell carcinoma of the lung are classified as NSCLC, there remains a degree of heterogeneity in their outcomes. Our findings indicate that squamous cell carcinoma of the lung tends to suppress the Mono perturbation response, whereas adenocarcinoma of the lung does not exhibit this phenomenon. This discrepancy may be attributed to the fact that monocytes play a specialized role in the development of lung adenocarcinoma. Within the tumor microenvironment, monocytes can differentiate into tumor-promoting M2-type macrophages or myeloid-derived suppressor cells (MDSCs), and they may also exert antitumor effects through specific subpopulations [58]. As highly plastic immune cells, monocytes display significant heterogeneity during tumorigenesis [58]. Further in-depth research is required to elucidate the precise biological mechanisms underlying these observations.

Our research inevitably faces several objective limitations. First, the GWAS dataset we utilized is predominantly derived from European populations. While this approach minimizes the impact of population heterogeneity, the generalizability of our findings to other populations should be considered with caution [59, 60]. Future research should aim to incorporate more diverse datasets to enhance the applicability of our results across a broader range of populations. Additionally, there is a possibility of overlapping subjects between exposure and outcome studies, which, despite F-statistic screening, may only partially avoidable. The number of SNPs retained after phenotype screening is relatively small, potentially introducing implicit biases. Furthermore, the limited sample size may contribute to additional bias. Non-random sample selection may bias single nucleotide polymorphism associations in various directions. For instance, phenotype-related selection can lead to attenuation bias, resulting in smaller estimated SNP effect sizes, potential false negatives, and smaller SNP heritabilities [61]. A larger dataset would provide more statistical power and help mitigate potential biases, thereby ensuring more reliable and robust results [61]. Furthermore, despite various tests conducted to ensure the robustness of the results, it is still difficult to avoid the inherent limitations of the MR method. Studies have shown that nearly half of the MR studies are sensitive to genetic pleiotropy to varying degrees, and the presence of unknown pathway bias can affect stability [23]. Another drawback of MR design is that it can only be applied to risk factors with suitable genetic variation, which inevitably leads to bias in the results [62]. Moreover, blood cell perturbation responses, as an exposure phenotype, can only be explained through genotypes. Bias can occur when the genotype is not randomly distributed within exposure subgroups, even if it is randomly distributed in the population [63, 64].

## Conclusion

This mendelian randomization study demonstrates causal associations between blood cell perturbation responses and lung cancer, identifying distinct protective (e.g., RET responses) and risk-associated phenotypes (e.g., RBC responses) across subtypes. These findings shed light on distinct biological mechanisms that could serve as valuable biomarkers or therapeutic targets, and underscore the complexity of hematological influences on lung cancer. These insights pave the way for innovative diagnostic and treatment approaches in lung cancer.

## Data Availability

All content used in this study is publicly available. The data that support this study are openly available in IEU Open GWAS project at https://gwas.mrcieu.ac.uk/. The summary statistics of GWAS related to blood cell perturbation responses are available in the GWAS catalog database (GCST90257015-GCST90257105).

## Abbreviations

CV: Coefficient of variation
EO: Eosinophils
FSC: Forward scatter
GWAS: Genome-wide association study
IPF: Immature platelet fraction
IVs: Instrumental variables
IVW: Inverse variance weighted
LUAD: Lung adenocarcinoma
LUSC: Squamous cell lung cancer
MDSCs: Myeloid-derived suppressor cells
Mono: Monocytes
MR: Mendelian randomization
NE: Neutrophils
NSCLC: Non-small cell lung carcinoma
OR: Odds ratio
OR_lci95: Lower 95% confidence interval odds ratio
OR_uci95: Upper 95% confidence interval odds ratio
PLT: Platelet
PLT-F: Platelet-F
Pval: P-value
RBC: Red blood cells
RET: Reticulocyte
ROS: Reactive oxygen species
SCLC: Small cell lung carcinoma
SD: Standard deviation
SE: Stress erythropoiesis
SFL: Side fluorescence
SNP: Single nucleotide polymorphism
SSC: Side scatter
UK: Unknown cell
WBC: White blood cell
WDF: White cell differential channel by fluorescence
WNR: White count and nucleated red blood cells
